# Diagnostic aid to rule out pneumonia in adults with cough and feeling of fever. A validation study in the primary care setting

**DOI:** 10.1186/1471-2334-12-355

**Published:** 2012-12-17

**Authors:** Ulrike Held, Claudia Steurer-Stey, Felix Huber, Sergio Dallafior, Johann Steurer

**Affiliations:** 1 Horten Center for Patient-oriented Research and Knowledge Transfer, University of Zurich, Postfach Nord, Pestalozzistrasse 24, Zurich CH-8091, Switzerland; 2 Institute of General Practice and Health Services Research, University of Zurich, Zurich, Switzerland; 3 Medix Gruppenpraxis, Rotbuchstrasse 46, Zurich, CH-8037, Switzerland; 4 Praxisgemeinschaft Altstetten, Eugen Huber-Strasse 16, 8048, Zürich, Switzerland

**Keywords:** Pneumonia, Primary care, Decision tree, Prescription of antibiotics, Validation study

## Abstract

**Background:**

We recently reported the derivation of a diagnostic aid to rule out pneumonia in adults presenting with new onset of cough or worsening of chronic cough and increased body temperature. The aim of the present investigation was to validate the diagnostic aid in a new sample of primary care patients.

**Methods:**

From two group practices in Zurich, we included 110 patients with the main symptoms of cough and subjective feeling of increased body temperature, and C-reactive protein levels below 50 μg/ml, no dyspnea, and not daily feeling of increased body temperature since the onset of cough. We excluded patients who were prescribed antibiotics at their first consultation. Approximately two weeks after inclusion, practice assistants contacted the participants by phone and asked four questions regarding the course of their complaints. In particular, they asked whether a prescription of antibiotics or hospitalization had been necessary within the last two weeks.

**Results:**

In 107 of 110 patients, pneumonia could be ruled out with a high degree of certainty, and no prescription of antibiotics was necessary. Three patients were prescribed antibiotics between the time of inclusion in the study and the phone interview two weeks later. Acute rhinosinusitis was diagnosed in one patient, and antibiotics were prescribed to the other two patients because their symptoms had worsened and their CRP levels increased. Use of the diagnostic aid could have missed these two possible cases of pneumonia. These observations correspond to a false negative rate of 1.8% (95% confidence interval: 0.50%-6.4%).

**Conclusions:**

This diagnostic aid is helpful to rule out pneumonia in patients from a primary care setting. After further validation application of this aid in daily practice may help to reduce the prescription rate of unnecessary antibiotics in patients with respiratory tract infections.

## Background

The inappropriate prescription of antibiotics is an avoidable cause of the increasing problem of antibiotic resistance. Overuse of antibiotics for upper respiratory tract infections is common, although guidelines recommend antibiotics only for patients with bacterial pneumonia or moderate to severe exacerbations of chronic bronchitis [1-4]. However, in a recent publication also a benefit of antibiotic treatment has been demonstrated in patients with mild to moderate exacerbations [5]. Physicians are aware of the recommendations in the guidelines, but they may have understandable concerns about missing pneumonia in patients with cough and increased body temperature, and when in doubt, physicians prefer to prescribe antibiotics.

In a recently published study [6] we reported the derivation of a fast and low-cost diagnostic aid to rule out pneumonia in patients with new onset of cough or worsening of chronic cough and the subjective feeling of increased body temperature. A total of 621 patients, almost all of them attending a primary care physician were included in the derivation study. The mean age of that patients was 48 years and half of them were males. The derivation study showed that three pieces of information are necessary to rule out pneumonia in these patients; C-reactive protein (CRP) level, dyspnea (no/yes), and daily feeling of increased body temperature since onset of cough (no/yes). Pneumonia could be ruled out in patients with CRP levels below 50 μg/ml who lacked dyspnea and daily subjective feeling of increased body temperature since onset of cough. At the time of publication, this diagnostic aid had not been validated in patients outside of the derivation sample.

Before integrating such a diagnostic aid into daily practice, the accuracy of the instrument should be tested in a new sample of patients. The aim of this study is to evaluate the accuracy of the diagnostic aid in patients with CRP levels below 50 μg/ml, no dyspnea and not daily subjective feeling of increased body temperature since onset of cough.

## Methods

The ethics committee of the Canton Zurich approved the study protocol, and we obtained informed consent from all participants.

### Recruitment of physicians

Physicians from two group practices in Zurich (mediX group practice and “Praxisgemeinschaft Altstetten,” both located in the urban Zurich area) were invited to participate in this study. They were provided with detailed information about the study, including the questionnaire for patient histories and the patient information leaflet.

### In- and exclusion criteria

Patients aged 16 years or older with the main symptom of new or worsened cough lasting at least 24 hours and who experienced the feeling of increased body temperature were potentially eligible for this study. Only patients in which our decision aid was negative, meaning patients with CRP levels less than 50 μg/ml (normal < 10 μg/ml), no dyspnea reported by the patient, no daily subjective feeling of increased body temperature since the onset of cough, and no treatment with antibiotics after the first consultation. We excluded patients with known chronic lung diseases (except chronic bronchitis), HIV-positive patients, patients who had taken oral corticosteroids within the last month, patients on chemotherapy, patients after organ transplantation, pregnant women, and patients incapable of reading the information leaflet and/or providing informed consent. Patients who were prescribed antibiotics at the initial consultation were excluded from the validation sample [7].

### Data gathering

Physicians assessed medical history after obtaining informed consent from the eligible patients. In particular, they asked patients about the presence of dyspnea and the duration and regularity (daily or not) subjective feeling of increased body temperature. Body temperature was not measured during consultation and patients were not instructed to measure body temperature at home. Venous blood samples for measuring CRP levels were drawn from all patients, and blood was analyzed using standard procedures. Other diagnostic procedures, treatment type, and the decision to initiate antimicrobial treatment were left to the discretion of the treating physician.

At a minimum of one week after the first consultation (inclusion of patients), physicians or physician assistants contacted the patients for follow-up only once by phone or during a planned office consultation. All patients were asked four questions: Have you received a prescription for antibiotics since the time of the consultation leading to inclusion in the study? Have you been hospitalized during this time? Has the severity of your cough decreased, increased, or remained stable since the first consultation? Has the subjective feeling of increased body temperature disappeared, remained stable, or worsened? When a patient had been hospitalized since inclusion in the study, we contacted hospitals to obtain the information whether pneumonia or another illness was the reason for the hospitalization.

When patients at the follow-up consultation (by phone or in the office) reported that symptoms improved and no antibiotics had been prescribed to them since the first consultation we assumed that a clinically relevant pneumonia could be excluded with a high degree of certainty.

#### Statistical analysis

The sample size of this study was planned to estimate the proportion of falsely classified non-pneumonia cases with a specified precision. For this we assumed p to be 2% (p = 0.02), and the standard error (p) = 0.015 such that the upper bound of the 95% confidence interval for p does not exceed 5%. These specifications led to a sample size of n = 88; we aimed to include at least 90 patients.

All patients fulfilling the inclusion criteria, and with complete answers to the four interview questions, were included in the statistical analysis. Descriptive statistics were calculated for patient demographic information, clinical signs of disease, and answers to the four interview questions. Categorical variables were displayed as percentages, and continuous variables as median and range. For the estimation of the proportion of falsely classified non-pneumonia cases $\hat{p}$, we determined the number of patients who received an antibiotics prescription for the diagnosis of pneumonia (question 1), and divided that number by n. We estimated the corresponding 95% confidence interval with the Wilson score interval [8]. All analyses were performed with R 2.14 [9].

## Results

Between December 2011 and May 2012, 115 patients were eligible for this study. Five patients who fulfilled the inclusion criteria were excluded because antibiotics had been prescribed during the first consultation. Therefore, 110 patients remained in the study for statistical analysis. To obtain information about the proportion of patients with cough and increased body temperature fulfill the inclusion criteria, the practice assistants registered all potentially eligible patients with cough and feeling of increased body temperature during two weeks in January 2012. Within those two weeks, the practice assistants counted 34 potentially eligible patients; ten of these patients, a bit less than a third, were eligible for inclusion in the study.

The median age of the included patients was 39 years (range 16 to 86 years), and 73 (66%) were female. The median duration between new onset and worsening of chronic cough was 7 days (interquartile range 4 to 14) days. The median CRP level was 6.3 μg/ml (range 1 to 44 μg/l), 61% of the included patients had a CRP level below 10 μg/l, and 39% of participants had a CRP level between 11 μg/l and 50 μg/l. In two patients, chest x-rays at the time of the first consultation showed no pathological changes. Detailed information about included patients is shown in Table 1.

**Table 1:** Baseline characteristics of 110 patients included in the study

**Variable**	**Statistic used**	**n = 110**
Baseline		
Age (years)	Median	39
	Range	16 - 86
	IQR	16.25
Gender	Male	37 (33.6%)
	Female	73 (66.4%)
Cough (since … days)	Median	7
	Range	2 - 75
	IQR	10
Chronic cough (> 4 weeks)		6 (5.5%)
CRP [μg/ml]	Median	6.3
	Range	0 - 44
	IQR	16.1
Chest x-ray	Yes	2 (1.8%)
	No	108 (98.2%)

All 110 patients enrolled in the study were contacted a median of 13 days (interquartile range 11 to 15 days) after enrollment in the study. Cough had improved in 90 (81.8%) of participants, had worsened in 6 (5.5%), and had remained stable in 11 (10%) of patients. Subjective feeling of increased body temperature disappeared completely in 98% of the patients, and two patients reported that they still had intermittent increased measured body temperature. In none of the patients a chest x-ray was performed after the first visit.

Three patients received a prescription for antibiotics between the time of inclusion in the study and the phone call by the practice assistant. For one patient with an increase of fever, deterioration of general health status, and an increase in CRP levels from 40 to 60 μg/ml two days after the first consultation, antibiotic treatment had been prescribed without an evaluative chest x-ray. In the second patient antibiotic treatment had been prescribed due to worsening of her cough and an increase in CRP level from 14 μg/ml to 67 μg/ml. No chest x-ray had been performed for this patient either. In the third patient a diagnosis of acute purulent rhinosinusitis was established three days after the first consultation, and treatment with antibiotics was initiated.

In 107 out of 110 patients, pneumonia could be ruled out with a very high degree of certainty. In the remaining three patients, who received antibiotics after inclusion, we did not miss an apparent case of pneumonia; there could have been one or two potential cases of pneumonia, but it is unlikely. Based on these data, the maximum proportion of missed pneumonia cases is $\hat{p}=2/110=0.018$ (95% confidence interval 0.005-0.064) as a worst-case scenario.

## Discussion

### Main results

We validated a simple diagnostic aid for ruling out pneumonia in patients with cough and increased body temperature [5] in a new primary care population. In patients with cough, feeling of increased body temperature, and a CRP level below 50 μg/ml, but without dyspnea and daily subjective feeling of increased body temperature since the onset of cough, pneumonia can be ruled out safely as the underlying illness. The predictive performance of the simple diagnostic aid was good, as no definite case of pneumonia was missed and only two potential cases of pneumonia appeared. If we rate both these cases as patients with missed pneumonia – the worst-case scenario − then the estimated proportion of falsely classified non-pneumonia cases is 1.8%.

### Limitations of the study

Patients included in this study represented approximately one-third of the patients attending general practitioners because of cough and subjective feeling of increased body temperature. The fact that no chest x-rays, the reference test, were performed in these patients may be seen as a limitation of this study [7]. It would not have been possible to obtain approval from the ethics committee to perform a chest x-ray in all patients. We therefore chose a pragmatic alternative to rule out “clinically relevant” pneumonia with a high degree of certainty by contacting patients approximately two weeks after the first consultation to ask them about the course of cough and subjective feeling of increased body temperature, prescription of antibiotics, and hospitalization. We cannot guarantee that we have not missed patients with pulmonary infiltrates that are consistent with pneumonia, but the probability that patients with moderate or severe pneumonia were missed is almost zero.

A further weakness of our study is that patients were probably not enrolled consecutively. We are not sure that all eligible patients with cough and increased body temperature were included in this study. The median duration of cough was one week, comparable to the duration of cough in the population of the derivation study [6]. This observation indicates that physicians may have preferentially included patients with longer duration of symptoms and, likely, a higher suspicion of pneumonia.

### Clinical implications

When physicians, after taking the patient history and performing the physical exam, are in doubt whether pneumonia could be present and therefore prescribe antibiotic treatment, the validated diagnostic aid is useful for two reasons. In patients with CRP levels below 50 μg/ml, no dyspnea, and no daily subjective feeling of increased body temperature since the onset of cough, pneumonia is very unlikely at the time of consultation. Unnecessary chest x-rays as well as unnecessary prescriptions of antibiotics can be avoided. However, symptoms could worsen in the days following the first consultation, making further tests and antibiotic treatment necessary.

Applying this diagnostic aid in daily practice may lead to a reduction in the overuse of antibiotics as well as a reduction in associated problems caused by this overuse. In our previous derivation study [7], we demonstrated that use of the simple diagnostic aid would have led to a decrease in antibiotic prescriptions of approximately ten percent even in a population of patients with a low risk of pneumonia. Acute respiratory tract infections are the most common reason for inappropriate antibiotic therapy in primary care. In view of the increasing problem of antibiotic resistance, possible side effects, and costs, even a small reduction is desirable and will impact public health.

### Implications for further research

In the future, the diagnostic aid should be validated in additional patient populations and in various health care settings. These validations will increase the reliability of this simple diagnostic instrument, and motivate physicians to apply it to their daily practice.

## Conclusion

Validation of the diagnostic aid in a new sample of primary care patients revealed that the instrument is highly accurate at ruling out pneumonia in patients with cough and subjective feeling of increased body temperature. The application of this rapid and low-cost instrument may support a change in physician behavior, reducing the rate of unnecessary chest x-rays and antibiotic prescriptions in patients with low risk of pneumonia. These improvements would markedly contribute to cost control and address the increasing problem of antibiotic resistance.

## Competing interests

The authors declare that they have no competing interests.

## Authors’ contributions

JS and UH developed the study protocol, were the study investigators, and drafted the manuscript. UH performed statistical analysis and interpreted the data. CS, FH, and SD were study investigators who contributed to and reviewed the drafts of the manuscript. All authors read and approved the final manuscript.

## Pre-publication history

The pre-publication history for this paper can be accessed here:

http://www.biomedcentral.com/1471-2334/12/355/prepub
